# Pathologic tonsillar findings similar to IgA nephropathy and the role of tonsillectomy in a patient with nephrotic syndrome

**DOI:** 10.1186/s12882-019-1580-y

**Published:** 2019-10-22

**Authors:** Takuji Enya, Tomoki Miyazawa, Kohei Miyazaki, Rina Oshima, Yuichi Morimoto, Mitsuru Okada, Tsukasa Takemura, Keisuke Sugimoto

**Affiliations:** 1 Department of Pediatrics, Kindai University Hospital, 377-2 Ohno-higashi, Osaka-Sayama, Osaka, 589-8511 Japan; 2 Department of Pediatrics, Kushimoto Municipality Faculty hospital, Wakayama, Japan

**Keywords:** Regulatory T cells, Self-tolerance, Frequently relapsing nephrotic syndrome, Tonsillectomy

## Abstract

**Abstract:**

Background: The pathological findings of tonsils in IgA nephropathy include the expansion of T-cell nodules around lymphoid follicles and abnormal reticulation of the crypt epithelium in contrast to chronic tonsillitis. Recently, several studies have reported that regulatory T cells play an important role in the maintenance of self-tolerance, an abnormality that is involved in the onset of nephrotic syndrome (NS). We encountered a patient of 28-year-old male with frequently relapsing nephrotic syndrome (FRNS) and chronic tonsillitis whose tonsils demonstrated pathological findings similar to those of IgA nephropathy.

**Case presentation:**

A patient had developed NS at the age of 5 years, and was pathologically diagnosed with minimal change disease (MCD), for which he received various immunosuppressive agents as treatment for recurrence. Because tonsillitis often triggers the recurrence of NS, a tonsillectomy was performed for chronic tonsillitis at the age of 25 years. Immunohistochemical staining of his tonsils showed the expansion of CD4 positive lymphocytes around the lymphoid follicles and abnormal reticulation of the crypt epithelium. The number of peripheral blood CD4^+^CD25^+^ regulatory T cells increased, and the frequency of relapses decreased after tonsillectomy.

**Conclusion:**

A similar self-tolerance abnormality exists in NS and IgA nephropathy; therefore, tonsillectomy might become a novel therapeutic approach for FRNS to redress the unbalanced self-tolerance and to remove the tonsillar focal infection. Further studies are necessary to verify the clinical efficiency of tonsillectomy for FRNS with recurrent episodes triggered by tonsillitis.

## Background

Regulatory T cells (Treg) are responsible for suppression of immune responses, and their function involves suppressing autoimmune diseases [1]. Treg are of critical importance for the maintenance of self-tolerance in IgA nephropathy [2]. In addition, several studies have reported that quantitative and qualitative abnormalities of Treg are involved in the pathophysiology of NS [3, 4]. Previously, IgA nephropathy was included in the focal inflammation-related diseases. Tonsillectomy along with steroid pulse therapy has been considered as one of the treatment strategies for IgA nephropathy in Asia [5], especially in Japan [6]. The pathological findings of the tonsils in IgA nephropathy differ significantly from those of chronic tonsillitis [7]. However, the pathological features of the tonsils in patients with NS are unknown. We encountered a patient with FRNS whose pathological findings of tonsils were similar to those of IgA nephropathy.

## Case presentation

The patient was a 28-year-old man who had developed NS at the age of 5 years. Renal biopsy was performed, and it showed no glomerular lesions. In addition, immunofluorescence (IF) disclosed no immunoglobulin and complement deposition. He was pathologically diagnosed with MCD, for which he received various immunosuppressive drugs including steroid pulse therapy, mizoribine, cyclosporine, cyclophosphamide, and mycophenolate mofetil for FRNS. However it had been difficult to maintain long-term remission. Twenty-one times of recurrences had occurred before having a tonsillectomy, and more than half of them were triggered by tonsillitis. He was diagnosed by an otolaryngologist with chronic tonsillitis, as he had four or more episodes of acute tonsillitis a year, and bilateral palatine tonsillectomy was performed for chronic tonsillitis to reduce the recurrence of tonsillitis. Informed consent for the operation was obtained from the patient. On admission, his height was 161.5 cm, body weight was 60.6 kg and blood pressure was 118/64 mmHg. No abnormality was observed on general physical examination. Urinalysis showed urine specific gravity of 1.021 and pH of 7.5. By urinary qualitative, urine protein and occult blood were negative. Urinary protein level was 0.02 g/day; β_2_-microglobulin was 139 μg/day. His blood urea nitrogen was 15.0 mg/dL, albumin was 4.0 g/dL, creatinine was 0.59 mg/dL, and cystatin C was 0.69 mg/L. Immunoglobulin and complement levels were normal.

**Fig. 1:**
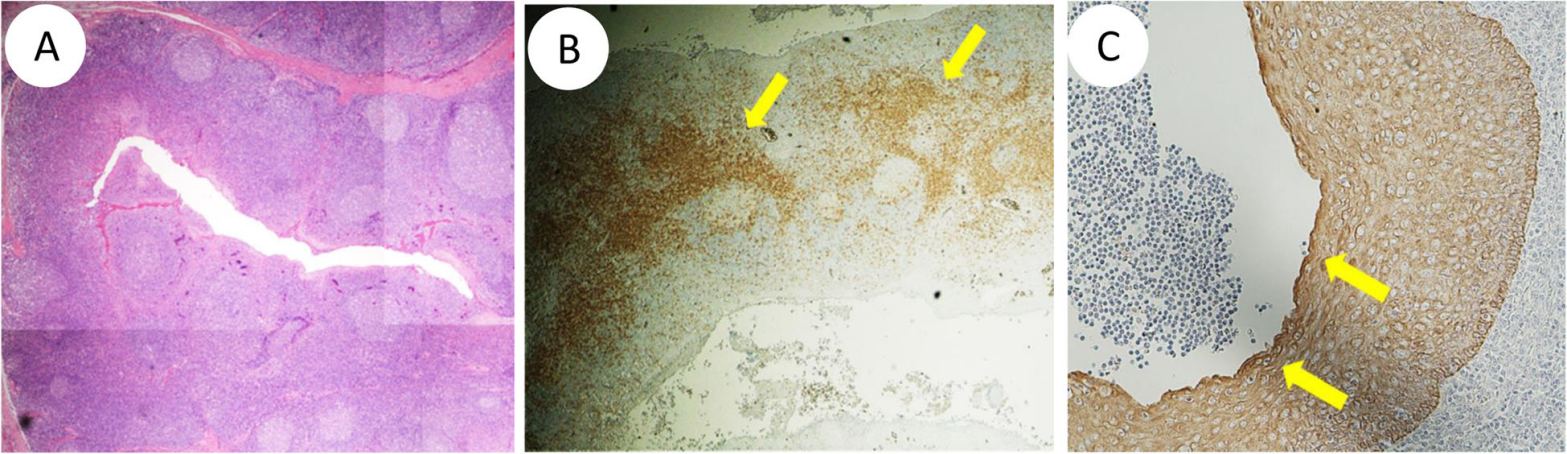
Pathological tonsillar findings in this patient. **a** The lymphoid follicles and germinal centers had various sizes. The boundary of each follicle was unclear and the expansion of the distance between follicles was observed. **b** CD4 staining showed T-cell nodules were enlarged by infiltration of CD4^+^ cells (allow). **c** Cytokeratin staining showed abnormal reticulation of the crypt epithelium (arrow)

The pathological findings of the tonsils are shown in Fig. 1. The lymphoid follicles and germinal centers were observed various sizes. The boundary of each follicle was unclear and the distance between follicles was expanded. T-cell nodules were enlarged due to infiltration of CD4^+^ cells. Abnormal reticulation of the crypt epithelium was observed by cytokeratin staining. Peripheral blood CD4 + CD25+ Treg count increased from 379/μL to 444/μL between 2 months pre and post tonsillectomy. After the tonsillectomy, the patient was followed up for 3 years, and the average number of recurrences of NS per year decreased from 1.1 times to 0.33 times.

## Discussion and conclusions

Tonsils are mucosa-associated lymphoid organs and tolerance regulating tissues involved in local immunity. Failure of this control system causes abnormalities of the immune reaction. Tonsillectomy for IgA nephropathy remains a controversial treatment. Kidney Disease Improving Global Outcomes (KDIGO) guidelines recommend tonsillectomy for IgA nephropathy with recurrent bacterial tonsillitis [8]. A meta-analysis of tonsillectomy for IgA nephropathy in Asia showed that tonsillectomy was effective with respect to clinical remission of proteinuria and renal protection [5]. The Treg counts correlatively decreased with high severity of IgA nephropathy; moreover, the Treg counts increased after tonsillectomy. Therefore, Treg may be involved in the pathophysiology of IgA nephropathy [2]. On the other hand, a few studies reported that the level of Treg expression in idiopathic NS was significantly lower than that of the control group [3, 9].

**Fig. 2:**
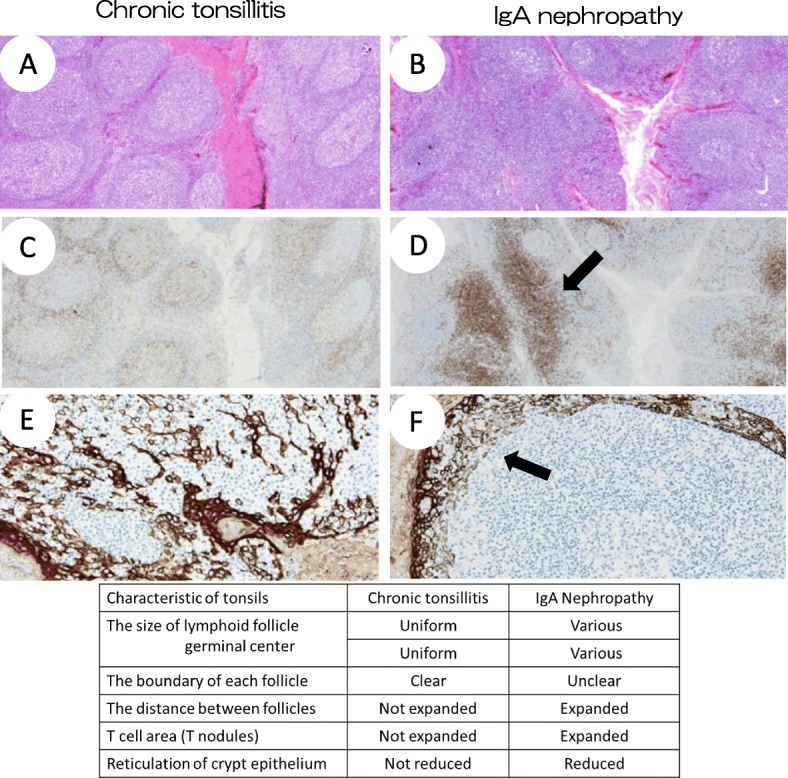
Differences of the histological structure of tonsils between chronic tonsillitis and IgA nephropathy. **a**, **b** Compared to chronic tonsillitis, in IgA nephropathy, the lymphoid follicle and germinal centers had various size. The boundary of each follicle was unclear and the distance between follicles was expanded. **c**, **d** In IgA nephropathy, T cell area was expanded by infiltration of CD4^+^ cells (allow). **e**, **f** In chronic tonsillitis, crypt epithelial cells exhibit a networked- structure by staining for cytokeratin. On the other hand, inhibition of crypt epithelial reticulation was observed in IgA nephropathy

Differences of the histological features of the palatine tonsils between chronic tonsillitis and IgA nephropathy are shown in Fig. 2. Crypt epithelial cells normally formed a networked- structure. Immune responses are initiated from antigen presentation to T-cells by lymphoepithelial symbiosis. However, immune responses caused by inhibiting the reticulation of the crypt epithelial cells are impaired and foreign antigens cannot be excluded efficiently in IgA nephropathy. As a result, chronic stimulation of tonsils by foreign antigens affects onset of IgA nephropathy [10, 11]. Since reduction of the reticulation of the crypt epithelium is affected by administration of corticosteroids, the possibility of the histological change due to long-term steroid therapy could not be denied. However, interestingly, the pathologic findings of the tonsils of patient with NS is similar to the findings of the tonsils of IgA nephropathy, and different from chronic tonsillitis. Furthermore, the peripheral blood CD4^+^CD25^+^ Treg counts increased after tonsillectomy. Treg are very important for the maintenance of self-tolerance and suppression of the immune system activation; hence, tonsillectomy may improve immune function. These findings suggested the involvement of Treg in NS; functional disorder of the local immune system might be associated with the pathophysiology of NS, including conditions such as IgA nephropathy. In routine medical practice, clinicians often experience the onset and recurrence of NS triggered by infection. Multiple factors could be associated with the onset of NS. However, tonsillectomy, by redressing the unbalanced self-tolerance, might be a novel therapeutic approach for FRNS with recurrent episodes triggered by tonsillitis.

We report a rare case of FRNS and chronic tonsillitis. The pathological findings of the tonsils in this case were similar to those of IgA nephropathy and tonsillectomy might be an optional therapeutic approach for FRNS that has not been effectively treated by existing therapy and with frequent recurrences triggered by tonsillitis. To our knowledge, there have been no reports on the role of tonsillectomy for NS and, further studies are necessary to verify the clinical efficacy of tonsillectomy. This could be of interest in a clinical scenario with respect to diagnosis and management in similar cases.

## Data Availability

The datasets during and/or analysed during the current study available from the corresponding author on reasonable request.

## Abbreviations

FRNS: Frequently relapsing nephrotic syndrome;
MCD: Minimal change disease
NS: Nephrotic syndrome
Treg: Regulatory T cells
