# Correction: Pathologic tonsillar findings similar to IgA nephropathy and the role of tonsillectomy in a patient with nephrotic syndrome

**DOI:** 10.1186/s12882-024-03529-7

**Published:** 2024-03-20

**Authors:** Takuji Enya, Tomoki Miyazawa, Kohei Miyazaki, Rina Oshima, Yuichi Morimoto, Mitsuru Okada, Tsukasa Takemura, Keisuke Sugimoto

**Affiliations:** 1 Department of Pediatrics, Kindai University Hospital Osaka-Sayama, 377-2 Ohno-higashi, 589–8511 Osaka, Japan; 2 Department of Pediatrics, Kushimoto Municipality Faculty hospital, Wakayama, Japan

**Correction: BMC Nephrology (2019) 20:381**

10.1186/s12882-019-1580-y.

Following publication of the original article [1], due to image ownership issues, the authors requested to remove Fig. 2 from this paper.
